# Glabridin

**DOI:** 10.1107/S1600536812048647

**Published:** 2012-11-30

**Authors:** Vimon Tantishaiyakul, Krit Suknuntha, Saowanit Saithong, Chaveng Pakawatchai

**Affiliations:** a Department of Pharmaceutical Chemistry, Faculty of Pharmaceutical Sciences, Prince of Songkla University and Nanotec-PSU Center of Excellence for Drug Delivery Systems, Hat-Yai, Songkhla 90112, Thailand; b Department of Chemistry and Center of Excellence for Innovation in Chemistry, Faculty of Science, Prince of Songkla University, Hat Yai, Songkhla 90112, Thailand

## Abstract

In the title compound, C_20_H_20_O_4_ {systematic name: 4-[(3*R*)-8,8-dimethyl-3,4-dihydro-2*H*-pyrano[2,3-*f*]chromen-3-yl]benz­ene-1,3-diol}, the hydro­pyran ring linked to the pendant benzene ring adopts an envelope conformation, with the methyne C atom forming the flap. In the crystal, the –OH group at the 3-position of the benzene ring forms an O—H⋯O hydrogen bond to a chromene O-atom acceptor, whereas the –OH group at the 1-position forms an O—H⋯π inter­action with a neighboring benzene ring. The O—H⋯O hydrogen bonds form [001] chains and the O—H⋯π bonds cross-link the chains into (101) sheets. The absolute structure was assumed to be the same as that deduced from previous studies for the natural product.

## Related literature

For background to the pharmacological activity of the title compound, see: Fukai *et al.* (2000 ▶); Messier & Grenier; (2011 ▶); Thiyagarajan *et al.* (2011 ▶); Ahn *et al.* (2012 ▶); Choi (2005 ▶). For the assignment of the absolute structure, see: Kim *et al.* (2009 ▶).
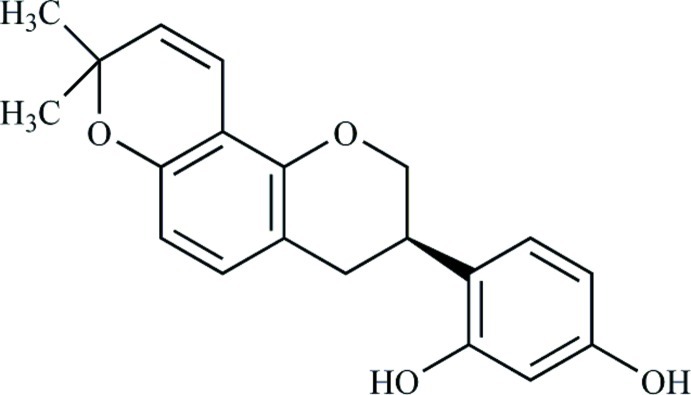


## Experimental

### 

#### Crystal data

C_20_H_20_O_4_
*M* *_r_* = 324.36
Orthorhombic, 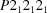

*a* = 6.4301 (4) Å
*b* = 12.0307 (7) Å
*c* = 21.0690 (13) Å
*V* = 1629.87 (17) Å^3^
*Z* = 4
Mo *K*α radiation
μ = 0.09 mm^−1^
*T* = 293 K
0.22 × 0.14 × 0.07 mm

#### Data collection

Bruker APEX CCD diffractometer
Absorption correction: multi-scan (*SADABS*; Bruker, 2003 ▶) *T*_min_ = 0.984, *T*_max_ = 0.994
15459 measured reflections
2866 independent reflections
2551 reflections with *I* > 2σ(*I*)
*R*_int_ = 0.045

#### Refinement

*R*[*F* ^2^ > 2σ(*F* ^2^)] = 0.046
*wR*(*F* ^2^) = 0.095
*S* = 1.16
2866 reflections
225 parameters
2 restraints
H atoms treated by a mixture of independent and constrained refinement
Δρ_max_ = 0.15 e Å^−3^
Δρ_min_ = −0.17 e Å^−3^

### 

Data collection: *SMART* (Bruker, 1998 ▶); cell refinement: *SAINT* (Bruker, 2003 ▶); data reduction: *SAINT*; program(s) used to solve structure: *SHELXS97* (Sheldrick, 2008 ▶); program(s) used to refine structure: *SHELXL97* (Sheldrick, 2008 ▶); molecular graphics: *Mercury* (Macrae *et al.*, 2008 ▶); software used to prepare material for publication: *SHELXTL* (Sheldrick, 2008 ▶) and *publCIF* (Westrip, 2010 ▶).

## Supplementary Material

Crystal structure: contains datablock(s) I, global. DOI: 10.1107/S1600536812048647/hb6989sup1.cif

Structure factors: contains datablock(s) I. DOI: 10.1107/S1600536812048647/hb6989Isup2.hkl

Supplementary material file. DOI: 10.1107/S1600536812048647/hb6989Isup3.cml

Additional supplementary materials:  crystallographic information; 3D view; checkCIF report

## Figures and Tables

**Table 1:** Hydrogen-bond geometry (Å, °) *Cg*4 is the centroid of the C13–C18 ring.

*D*—H⋯*A*	*D*—H	H⋯*A*	*D*⋯*A*	*D*—H⋯*A*
O4—H4*A*⋯O1^i^	0.82 (2)	2.02 (2)	2.841 (3)	177 (3)
O3—H3*A*⋯*Cg*4^ii^	0.80 (2)	2.51 (2)	3.213 (2)	148 (3)

Symmetry codes: (i) 
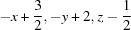
; (ii) 
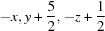
.

**Fig. 1.**
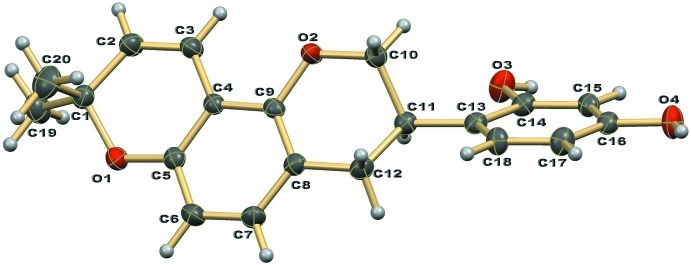
The molecular structure of the title compound.

**Fig. 2.**
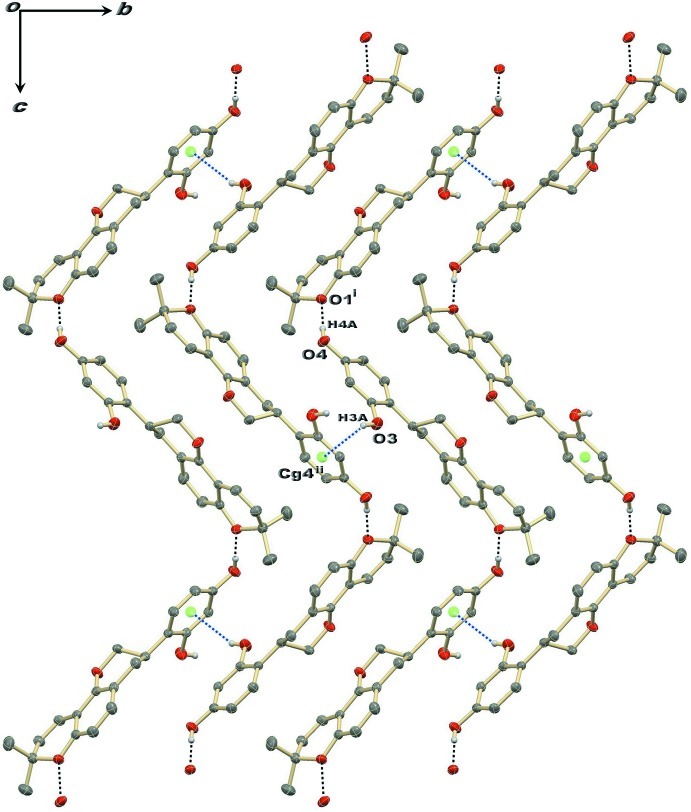
The packing of the intermolecular interactions of the title compound is plotted down a axis.

## supplementary crystallographic information

### Comment

4-[(3*R*)-8,8-Dimethyl-3,4-dihydro-2*H*-pyrano[2,3-*f*]chromen-3-yl]benzene-1,3-diol (Glabridin) is a pyranoisoflavan isolated from licorice. It has various pharmacological activities such as cytotoxic activity (Fukai *et al.*, 2000), antimicrobial activity (Messier & Grenier, 2011), anti-inflammation (Thiyagarajan *et al.*, 2011), anti-obesity effect (Ahn *et al.*, 2012) and prevention for osteoporosis and inflammatory bone diseases (Choi, 2005). For the assignment of its absolute structure, see: Kim *et al.* (2009).

The molecular structure of the title compound is shown in Fig. 1. The packing features O—H···O intermolecular hydrogen bonding between hydroxyl group at 3 position of benzene ring with the donor-acceptor distance of 2.841 (3) Å. (O4—H4A···O1^i^; i: -*x* + 3/2, -*y* + 2, *z* - 1/2) forming a zigzag chains running parallel to the [001] direction. Besides, the O—H···π interactions with O···centroid distances of 3.213 (2) Å are observed between hydroxyl group at the 1 position of benzene ring and the nearby benzene ring of adjacent molecule, O3—H3A···*Cg*4^ii^ (*Cg*4 is the centroid of C13—C14—C15—C16—C17—C18 and the symmetry code ii is -*x*, *y* + 5/2, -*z* + 1/2), linking among the zigzag chains generating two-dimensional layer parallel to (101) plane. The crystal packing of interaction is depicted in Fig. 2.

### Experimental

The title compound was obtained from Nanjing Zelang Medical Technology Co. Ltd. Colourless blocks were obtained by dissolving the compound in methanol followed by a slow evaporation of the solvent.

### Refinement

Amonalous dispersion was found to be negligible and the absolute structure is indeterminate. Friedel pairs were merged before the final refinement. H atoms on carbon atoms were positioned geometrically and constrained to ride on their parent atoms with *U*_iso_(H) = 1.2*U*_eq_(C-*sp*^2^) with C—H = 0.93 Å and 1.5*U*_eq_(C-*sp*^3^) with the distances ranging from 0.96 to 0.98 Å, respectively. The H atoms on the oxygen atoms were located in a difference Fourier map and restrained with *U*_iso_(H) = 1.2*U*_eq_(OH). (O—H = 0.80 (2) and 0.82 (2) Å).

### Crystal data

**Table:**

C_20_H_20_O_4_	*F*(000) = 688
*M**_r_* = 324.36	*D*_x_ = 1.322 Mg m^−^^3^
Orthorhombic, *P*2_1_2_1_2_1_	Mo *K*α radiation, λ = 0.71073 Å
Hall symbol: P 2ac 2ab	Cell parameters from 2104 reflections
*a* = 6.4301 (4) Å	θ = 3.3–21.0°
*b* = 12.0307 (7) Å	µ = 0.09 mm^−^^1^
*c* = 21.0690 (13) Å	*T* = 293 K
*V* = 1629.87 (17) Å^3^	Block, colourless
*Z* = 4	0.22 × 0.14 × 0.07 mm

### Data collection

**Table:**

Bruker APEX CCD diffractometer	2866 independent reflections
Radiation source: fine-focus sealed tube	2551 reflections with *I* > 2σ(*I*)
Graphite monochromator	*R*_int_ = 0.045
Frames, each covering 0.3 ° in ω scans	θ_max_ = 25.0°, θ_min_ = 1.9°
Absorption correction: multi-scan (*SADABS*; Bruker, 2003)	*h* = −7→7
*T*_min_ = 0.984, *T*_max_ = 0.994	*k* = −14→14
15459 measured reflections	*l* = −25→25

### Refinement

**Table:**

Refinement on *F*^2^	Primary atom site location: structure-invariant direct methods
Least-squares matrix: full	Secondary atom site location: difference Fourier map
*R*[*F*^2^ > 2σ(*F*^2^)] = 0.046	Hydrogen site location: inferred from neighbouring sites
*wR*(*F*^2^) = 0.095	H atoms treated by a mixture of independent and constrained refinement
*S* = 1.16	*w* = 1/[σ^2^(*F*_o_^2^) + (0.0365*P*)^2^ + 0.2296*P*] where *P* = (*F*_o_^2^ + 2*F*_c_^2^)/3
2866 reflections	(Δ/σ)_max_ < 0.001
225 parameters	Δρ_max_ = 0.15 e Å^−^^3^
2 restraints	Δρ_min_ = −0.17 e Å^−^^3^

### Special details

**Table:**

Geometry. All e.s.d.'s (except the e.s.d. in the dihedral angle between two l.s. planes) are estimated using the full covariance matrix. The cell e.s.d.'s are taken into account individually in the estimation of e.s.d.'s in distances, angles and torsion angles; correlations between e.s.d.'s in cell parameters are only used when they are defined by crystal symmetry. An approximate (isotropic) treatment of cell e.s.d.'s is used for estimating e.s.d.'s involving l.s. planes.
Refinement. Refinement of *F*^2^ against all reflections. The weighted *R*-factor *wR* and goodness of fit *S* are based on *F*^2^, conventional *R*-factors *R* are based on *F*, with *F* set to zero for negative *F*^2^. The threshold expression of *F*^2^ > 2*σ*(*F*^2^) is used only for calculating *R*-factors(gt) *etc*. and is not relevant to the choice of reflections for refinement. *R*-factors based on *F*^2^ are statistically about twice as large as those based on *F*, and *R*- factors based on all data will be even larger.

### Fractional atomic coordinates and isotropic or equivalent isotropic displacement parameters (Å^2^)

**Table:**

	*x*	*y*	*z*	*U*_iso_*/*U*_eq_	
O1	0.7767 (3)	0.66156 (13)	0.23880 (7)	0.0443 (4)	
O2	1.0695 (3)	0.80853 (13)	0.05050 (7)	0.0477 (5)	
C1	0.9085 (4)	0.56254 (19)	0.23598 (11)	0.0417 (6)	
C2	1.1009 (4)	0.5878 (2)	0.19907 (11)	0.0458 (6)	
H2	1.2221	0.5486	0.2080	0.055*	
C3	1.1042 (4)	0.6644 (2)	0.15409 (11)	0.0424 (6)	
H3	1.2243	0.6754	0.1303	0.051*	
C4	0.9211 (4)	0.73112 (18)	0.14167 (10)	0.0326 (5)	
C5	0.7586 (4)	0.72632 (18)	0.18500 (10)	0.0370 (6)	
C6	0.5850 (4)	0.7914 (2)	0.17877 (12)	0.0479 (6)	
H6	0.4783	0.7876	0.2085	0.057*	
C7	0.5712 (4)	0.8631 (2)	0.12745 (12)	0.0466 (6)	
H7	0.4530	0.9070	0.1230	0.056*	
C8	0.7277 (4)	0.87146 (18)	0.08254 (11)	0.0366 (5)	
C9	0.9013 (4)	0.80460 (18)	0.09051 (10)	0.0340 (5)	
C10	1.0643 (4)	0.88701 (19)	−0.00121 (10)	0.0431 (6)	
H10A	1.0019	0.8517	−0.0380	0.052*	
H10B	1.2055	0.9076	−0.0123	0.052*	
C11	0.9426 (4)	0.99118 (18)	0.01485 (10)	0.0357 (5)	
H11	1.0017	1.0217	0.0540	0.043*	
C12	0.7201 (4)	0.95505 (19)	0.02970 (12)	0.0435 (6)	
H12A	0.6376	1.0188	0.0424	0.052*	
H12B	0.6567	0.9223	−0.0076	0.052*	
C13	0.9633 (4)	1.07917 (18)	−0.03561 (10)	0.0353 (5)	
C14	1.1391 (4)	1.14759 (18)	−0.03656 (10)	0.0364 (6)	
C15	1.1636 (4)	1.23098 (19)	−0.08114 (11)	0.0378 (5)	
H15	1.2811	1.2760	−0.0802	0.045*	
C16	1.0135 (4)	1.24727 (18)	−0.12703 (10)	0.0372 (6)	
C17	0.8386 (4)	1.1812 (2)	−0.12775 (11)	0.0449 (6)	
H17	0.7366	1.1918	−0.1585	0.054*	
C18	0.8159 (4)	1.09882 (19)	−0.08240 (11)	0.0425 (6)	
H18	0.6970	1.0549	−0.0833	0.051*	
C19	0.9574 (5)	0.5352 (3)	0.30460 (12)	0.0639 (8)	
H19A	1.0368	0.5946	0.3230	0.096*	
H19B	1.0363	0.4675	0.3065	0.096*	
H19C	0.8300	0.5261	0.3278	0.096*	
C20	0.7812 (6)	0.4713 (2)	0.20419 (15)	0.0721 (9)	
H20A	0.6555	0.4593	0.2278	0.108*	
H20B	0.8608	0.4038	0.2031	0.108*	
H20C	0.7472	0.4934	0.1617	0.108*	
O3	1.2858 (3)	1.12753 (14)	0.00917 (9)	0.0536 (5)	
H3A	1.362 (4)	1.1797 (18)	0.0133 (14)	0.064*	
O4	1.0477 (3)	1.33015 (16)	−0.16975 (8)	0.0551 (5)	
H4A	0.957 (4)	1.334 (2)	−0.1969 (11)	0.066*	

### Atomic displacement parameters (Å^2^)

**Table:**

	*U*^11^	*U*^22^	*U*^33^	*U*^12^	*U*^13^	*U*^23^
O1	0.0529 (11)	0.0443 (9)	0.0356 (9)	0.0025 (8)	0.0083 (8)	0.0037 (8)
O2	0.0485 (11)	0.0493 (10)	0.0452 (9)	0.0166 (9)	0.0169 (9)	0.0160 (8)
C1	0.0460 (15)	0.0360 (13)	0.0431 (13)	−0.0006 (11)	0.0002 (13)	0.0026 (11)
C2	0.0429 (16)	0.0503 (15)	0.0443 (14)	0.0106 (13)	0.0013 (12)	0.0064 (12)
C3	0.0391 (14)	0.0493 (14)	0.0389 (13)	0.0042 (12)	0.0071 (12)	0.0038 (12)
C4	0.0349 (12)	0.0318 (11)	0.0312 (11)	−0.0027 (10)	0.0009 (11)	−0.0031 (10)
C5	0.0418 (14)	0.0341 (12)	0.0351 (12)	−0.0048 (11)	0.0001 (11)	−0.0035 (10)
C6	0.0435 (15)	0.0524 (15)	0.0479 (14)	0.0039 (13)	0.0142 (13)	0.0032 (12)
C7	0.0337 (14)	0.0463 (14)	0.0598 (15)	0.0073 (12)	0.0054 (13)	0.0070 (13)
C8	0.0328 (13)	0.0336 (12)	0.0433 (13)	−0.0014 (11)	−0.0025 (12)	−0.0027 (11)
C9	0.0347 (13)	0.0339 (11)	0.0335 (12)	−0.0036 (10)	0.0048 (11)	−0.0061 (10)
C10	0.0521 (15)	0.0446 (13)	0.0326 (12)	0.0064 (12)	0.0058 (12)	0.0053 (11)
C11	0.0381 (13)	0.0372 (12)	0.0319 (12)	0.0005 (11)	−0.0050 (11)	−0.0018 (10)
C12	0.0403 (15)	0.0419 (13)	0.0482 (14)	0.0067 (12)	−0.0014 (12)	0.0038 (12)
C13	0.0405 (14)	0.0350 (12)	0.0304 (12)	0.0018 (11)	−0.0035 (11)	−0.0038 (10)
C14	0.0383 (14)	0.0353 (12)	0.0355 (12)	0.0014 (11)	−0.0093 (11)	−0.0044 (11)
C15	0.0370 (14)	0.0352 (12)	0.0413 (12)	−0.0063 (11)	−0.0006 (11)	−0.0031 (11)
C16	0.0463 (15)	0.0352 (12)	0.0302 (12)	0.0006 (12)	−0.0021 (11)	−0.0016 (10)
C17	0.0506 (16)	0.0469 (14)	0.0372 (13)	−0.0027 (13)	−0.0152 (12)	0.0017 (12)
C18	0.0447 (15)	0.0426 (13)	0.0402 (13)	−0.0106 (12)	−0.0083 (13)	0.0005 (12)
C19	0.067 (2)	0.076 (2)	0.0484 (16)	0.0029 (18)	0.0022 (16)	0.0185 (15)
C20	0.086 (2)	0.0478 (16)	0.082 (2)	−0.0106 (18)	−0.008 (2)	−0.0059 (16)
O3	0.0471 (11)	0.0522 (11)	0.0616 (11)	−0.0086 (9)	−0.0261 (10)	0.0097 (10)
O4	0.0654 (13)	0.0539 (11)	0.0462 (10)	−0.0134 (11)	−0.0119 (9)	0.0168 (9)

### Geometric parameters (Å, º)

**Table:**

O1—C5	1.380 (3)	C11—C12	1.528 (3)
O1—C1	1.463 (3)	C11—H11	0.9800
O2—C9	1.372 (3)	C12—H12A	0.9700
O2—C10	1.442 (2)	C12—H12B	0.9700
C1—C2	1.493 (4)	C13—C18	1.388 (3)
C1—C19	1.516 (3)	C13—C14	1.398 (3)
C1—C20	1.524 (4)	C14—O3	1.369 (3)
C2—C3	1.321 (3)	C14—C15	1.383 (3)
C2—H2	0.9300	C15—C16	1.380 (3)
C3—C4	1.449 (3)	C15—H15	0.9300
C3—H3	0.9300	C16—O4	1.361 (3)
C4—C5	1.389 (3)	C16—C17	1.377 (3)
C4—C9	1.400 (3)	C17—C18	1.384 (3)
C5—C6	1.370 (3)	C17—H17	0.9300
C6—C7	1.386 (3)	C18—H18	0.9300
C6—H6	0.9300	C19—H19A	0.9600
C7—C8	1.385 (3)	C19—H19B	0.9600
C7—H7	0.9300	C19—H19C	0.9600
C8—C9	1.386 (3)	C20—H20A	0.9600
C8—C12	1.501 (3)	C20—H20B	0.9600
C10—C11	1.516 (3)	C20—H20C	0.9600
C10—H10A	0.9700	O3—H3A	0.802 (17)
C10—H10B	0.9700	O4—H4A	0.820 (17)
C11—C13	1.506 (3)		
			
C5—O1—C1	118.37 (17)	C13—C11—H11	107.3
C9—O2—C10	117.92 (18)	C10—C11—H11	107.3
O1—C1—C2	109.58 (18)	C12—C11—H11	107.3
O1—C1—C19	105.0 (2)	C8—C12—C11	108.17 (19)
C2—C1—C19	111.7 (2)	C8—C12—H12A	110.1
O1—C1—C20	107.1 (2)	C11—C12—H12A	110.1
C2—C1—C20	111.3 (2)	C8—C12—H12B	110.1
C19—C1—C20	112.0 (2)	C11—C12—H12B	110.1
C3—C2—C1	121.9 (2)	H12A—C12—H12B	108.4
C3—C2—H2	119.0	C18—C13—C14	116.2 (2)
C1—C2—H2	119.0	C18—C13—C11	124.1 (2)
C2—C3—C4	120.2 (2)	C14—C13—C11	119.7 (2)
C2—C3—H3	119.9	O3—C14—C15	121.8 (2)
C4—C3—H3	119.9	O3—C14—C13	116.3 (2)
C5—C4—C9	117.7 (2)	C15—C14—C13	121.9 (2)
C5—C4—C3	118.02 (19)	C16—C15—C14	119.9 (2)
C9—C4—C3	124.2 (2)	C16—C15—H15	120.0
C6—C5—O1	118.1 (2)	C14—C15—H15	120.0
C6—C5—C4	121.8 (2)	O4—C16—C17	123.2 (2)
O1—C5—C4	120.0 (2)	O4—C16—C15	117.0 (2)
C5—C6—C7	118.9 (2)	C17—C16—C15	119.8 (2)
C5—C6—H6	120.6	C16—C17—C18	119.5 (2)
C7—C6—H6	120.6	C16—C17—H17	120.3
C8—C7—C6	122.1 (2)	C18—C17—H17	120.3
C8—C7—H7	118.9	C17—C18—C13	122.7 (2)
C6—C7—H7	118.9	C17—C18—H18	118.7
C7—C8—C9	117.4 (2)	C13—C18—H18	118.7
C7—C8—C12	122.1 (2)	C1—C19—H19A	109.5
C9—C8—C12	120.3 (2)	C1—C19—H19B	109.5
O2—C9—C8	122.7 (2)	H19A—C19—H19B	109.5
O2—C9—C4	115.1 (2)	C1—C19—H19C	109.5
C8—C9—C4	122.2 (2)	H19A—C19—H19C	109.5
O2—C10—C11	112.64 (17)	H19B—C19—H19C	109.5
O2—C10—H10A	109.1	C1—C20—H20A	109.5
C11—C10—H10A	109.1	C1—C20—H20B	109.5
O2—C10—H10B	109.1	H20A—C20—H20B	109.5
C11—C10—H10B	109.1	C1—C20—H20C	109.5
H10A—C10—H10B	107.8	H20A—C20—H20C	109.5
C13—C11—C10	112.19 (18)	H20B—C20—H20C	109.5
C13—C11—C12	115.3 (2)	C14—O3—H3A	111 (2)
C10—C11—C12	107.10 (19)	C16—O4—H4A	113 (2)
			
C5—O1—C1—C2	40.3 (3)	C3—C4—C9—O2	1.3 (3)
C5—O1—C1—C19	160.4 (2)	C5—C4—C9—C8	0.3 (3)
C5—O1—C1—C20	−80.5 (3)	C3—C4—C9—C8	−176.3 (2)
O1—C1—C2—C3	−28.0 (3)	C9—O2—C10—C11	31.4 (3)
C19—C1—C2—C3	−143.8 (3)	O2—C10—C11—C13	171.8 (2)
C20—C1—C2—C3	90.2 (3)	O2—C10—C11—C12	−60.7 (3)
C1—C2—C3—C4	3.5 (4)	C7—C8—C12—C11	147.1 (2)
C2—C3—C4—C5	11.3 (3)	C9—C8—C12—C11	−28.3 (3)
C2—C3—C4—C9	−172.2 (2)	C13—C11—C12—C8	−177.54 (18)
C1—O1—C5—C6	156.1 (2)	C10—C11—C12—C8	56.8 (2)
C1—O1—C5—C4	−29.1 (3)	C10—C11—C13—C18	99.2 (3)
C9—C4—C5—C6	−0.6 (3)	C12—C11—C13—C18	−23.7 (3)
C3—C4—C5—C6	176.2 (2)	C10—C11—C13—C14	−81.4 (3)
C9—C4—C5—O1	−175.14 (19)	C12—C11—C13—C14	155.7 (2)
C3—C4—C5—O1	1.7 (3)	C18—C13—C14—O3	−179.3 (2)
O1—C5—C6—C7	175.3 (2)	C11—C13—C14—O3	1.2 (3)
C4—C5—C6—C7	0.6 (4)	C18—C13—C14—C15	0.7 (3)
C5—C6—C7—C8	−0.4 (4)	C11—C13—C14—C15	−178.7 (2)
C6—C7—C8—C9	0.2 (4)	O3—C14—C15—C16	179.0 (2)
C6—C7—C8—C12	−175.3 (2)	C13—C14—C15—C16	−1.1 (3)
C10—O2—C9—C8	0.9 (3)	C14—C15—C16—O4	−179.5 (2)
C10—O2—C9—C4	−176.63 (19)	C14—C15—C16—C17	0.8 (3)
C7—C8—C9—O2	−177.5 (2)	O4—C16—C17—C18	−179.9 (2)
C12—C8—C9—O2	−1.9 (3)	C15—C16—C17—C18	−0.2 (3)
C7—C8—C9—C4	−0.2 (3)	C16—C17—C18—C13	−0.2 (4)
C12—C8—C9—C4	175.5 (2)	C14—C13—C18—C17	−0.1 (3)
C5—C4—C9—O2	177.88 (19)	C11—C13—C18—C17	179.3 (2)

### Hydrogen-bond geometry (Å, º)

Cg4 is the centroid of the C13–C18 ring.

**Table:**

*D*—H···*A*	*D*—H	H···*A*	*D*···*A*	*D*—H···*A*
O4—H4*A*···O1^i^	0.82 (2)	2.02 (2)	2.841 (3)	177 (3)
O3—H3*A*···*Cg*4^ii^	0.80 (2)	2.51 (2)	3.213 (2)	148 (3)

Symmetry codes: (i) −*x*+3/2, −*y*+2, *z*−1/2; (ii) −*x*, *y*+5/2, −*z*+1/2.
